# Activated Toxicity of Diesel Particulate Extract by Ultraviolet A Radiation in Mammalian Cells: Role of Singlet Oxygen

**DOI:** 10.1289/ehp.0800029

**Published:** 2008-09-15

**Authors:** Lingzhi Bao, An Xu, Liping Tong, Shaopeng Chen, Lingyan Zhu, Ye Zhao, Guoping Zhao, Erkang Jiang, Jun Wang, Lijun Wu

**Affiliations:** Key Laboratory of Ion Beam Bioengineering, Institute of Plasma Physics, Chinese Academy of Sciences, Hefei, Anhui, People’s Republic of China

**Keywords:** A_L_ cell, cytotoxicity, diesel particulate extracts, genotoxicity, singlet oxygen, UVA

## Abstract

**Background:**

Diesel exhaust [diesel exhaust particles (DEPs) and their extracts (DPE)] and ultraviolet A radiation (UVA) are two ubiquitous environmental factors that have been identified as essential risk factors for various benign or malignant human diseases, either alone or in combination with other agents.

**Objectives:**

We aimed to investigate the synergistic effects of DPE and UVA at low-dose exposures in human–hamster hybrid (A_L_) cells and their underlying mechanisms.

**Methods:**

We exposed exponentially growing A_L_ cells to DPE and/or UVA radiation with or without reactive oxygen species (ROS) quenchers and then assayed the cells for survival, mutation induction, apoptosis, and micronucleus generation. In addition, using a singlet oxygen (1O_2_) trapping probe, 2,2,6,6-tetramethyl-4-piperidone, coupled with electron paramagnetic resonance spectroscopy, we determined the production of 1O_2_.

**Results:**

Treatment of A_L_ cells with DPE + UVA induced significant cytotoxic and genotoxic damage. In contrast, we found no significant damage in cells treated with either UVA or DPE alone at the same doses. Mutation spectra of *CD59*^−^ mutants showed that treatment with DPE + UVA easily induces multilocus deletions. Sodium azide significantly inhibited both cellular and DNA damage induced by DPE + UVA treatment, whereas other ROS inhibitors had little protecting effect. Furthermore, we found a significant increase of 1O_2_ in the cells that received DPE + UVA treatment.

**Conclusion:**

These findings suggest that UVA activated the genotoxicity and cytotoxicity of DPE in mammalian cells and that 1O_2_ played an important role in these processes.

The popularity of diesel engines has been steadily increasing recently because of fuel efficiency, longevity, high torque at highway speeds, safety, and economy concerns (Lloyd and Cackette 2001). Diesel exhaust emitted at ground level is generated during the combustion process and consists of hundreds of organic and inorganic compounds in either gaseous or particulate phases (Kagawa 2002). The dominant pollutant in ambient air, diesel exhaust particles (DEPs) consist of inert carbonaceous cores with large surface areas. This property is ideal for the adsorption of trace transition metals and various organic substances, including polycyclic aromatic hydrocarbons (PAHs), nitroaromatic hydrocarbons, quinine, and acids (McClellan 1987). Several national and international agencies have classified DEPs as a “potential” or “probable” human carcinogen [International Agency for Research on Cancer (IARC) 1989]. DEP exposure causes DNA and chromosomal damage, including bulky DNA adducts, oxidized bases, deletions, and chromosomal aberrations, which may lead to a broad spectrum of mutations (DeMarini et al. 2004; Müller et al. 2004; Tsurudome et al. 1999). Recent evidence has demonstrated that DEP and diesel particle extracts (DPEs) are highly mutagenic to TA98 and TA100 *Salmonella* strains in the Ames test and induce a dose-dependent increase in mutation yield that is suppressed by the S9 mixture (DeMarini et al. 2004). However, results at the hypoxanthine-guanine phosphoribosyl transferase (*Hprt*) locus in mammalian cells and the λ/*lacI* locus in transgenic mice varied in different studies (Dybdahl et al. 2004; Gu et al. 2005; Risom et al. 2003; Sato et al. 2000).

Ultraviolet (UV) radiation from sunlight, including UVA and UVB, is a major factor for causing skin aging, skin cancer, and cytogenetic damage in lung, bone marrow, and peripheral blood erythrocytes (Balansky et al. 2003; Placzek et al. 2004; Yin et al. 2001). UVB (280–320 nm) is directly absorbed by DNA and induces damage, such as cyclobutane pyrimidine dimers and pyrimidine (6-4)pyrimidone photoproducts (de Gruijl 2000; Ravanat et al. 2001), which may contribute to the carcinogenicity of UVB. In contrast, UVA (320–400 nm), the major component of solar UV radiation, is considered to be less carcinogenic than UVB because the DNA absorption of UVA is extremely weak. However, recent evidence shows that UVA also induces various forms of DNA damage in the presence of either endogenous or exogenous photosensitizers, such as PAHs like benzo[*a*]pyrene (Kawanishi and Hiraku 2001; Ravanat et al. 2001). The toxicity of exposure to PAHs plus UV has been observed in laboratory animals (Cavalieri and Calvin 1971; Clark 1964; Stenbäck 1975) and various cells (Kagan et al. 1989; Schirmer et al. 1998; Utesch et al. 1996). More recently, several groups have demonstrated that coexposure to PAHs and UVA significantly augmented DNA damage, such as single-strand breaks (Dong et al. 2000), double-strand breaks (Toyooka et al. 2004, 2006), and the formation of 8-hydroxy-2′-deoxyguanosine, (8-oxodG; Mauthe et al. 1995), which are closely associated with mutation and carcinogenesis. UV or sunlight exposure per se as well as in combination with other agents has been identified as a risk factor for various benign or malignant human diseases. For example, combined exposure to tobacco smoke and sunlight is associated with dysplastic and malignant lip lesions and squamous cell carcinoma of the skin (King et al. 1995; Vander Straten et al. 2001). In aquatic organisms, the toxicity of PAHs increased considerably when combined with UV radiation (Boese et al. 2000; Laycock et al. 2000; Nikkilä et al. 1999). Exposure of organic extracts of air particulates to sunlight was also found to lead to an increase in mutagenicity in a *Salmonella* assay (al-Khodairy and Hannan 1997).

Diesel exhaust and UV radiation are two ubiquitous environmental factors. Individuals such as road maintenance workers and traffic policemen are easily exposed to these two factors simultaneously in their daily lives. Although the exposure to either diesel exhaust or UVA radiation alone or in combination with other agents has been identified as essential risk factors for various benign or malignant human diseases (King et al. 1995; Morgan et al. 1997; Siegel et al. 2004; Vander Straten et al. 2001), the synergistic effects of DPE and UVA have not been clearly clarified. In the present study, we focused on the cytotoxicity and genotoxicity of either DPE or UVA exposure alone or simultaneously in a human–hamster hybrid (A_L_) cell line. Our results indicated the high risk of coexposure to diesel exhaust and sunlight, which might be mediated by singlet oxygen (1O_2_).

## Materials and Methods

### Cell culture

In this study, we used the A_L_ cell line, a human–hamster hybrid formed by fusion of human fibroblasts and the gly2A mutant of Chinese hamster ovary (CHO) cells (Ueno et al. 1996). In addition to a standard set of CHO-K1 chromosomes, these hybrid cells contain a single copy of human chromosome 11, which encodes several cell-surface antigenic markers that render the cells sensitive to killing by specific monoclonal antibodies in the presence of rabbit serum complement (HPR, Inc., Denver, PA, USA). This cell line is sensitive in detecting mutagens that induce mostly large, multilocus deletions such as ionizing radiation, asbestos fibers, and certain heavy metals (Liu et al. 2005; Ueno et al. 1996; Xu et al. 2002). Because only a small segment of the human chromosome (11p15.5) is required for the viability of A_L_ cells, mutations based on marker genes located in the human chromosome ranging in size up to 140 Mbp of DNA can be detected (Wilson et al. 1999).

We cultured cells in Ham’s F-12 medium (Gibco, Grand Island, NY, USA) supplemented with 8% heated inactivated fetal bovine serum (Hyclone, Grand Island, NY, USA), 2 × 10^−4^ M glycine, and 25 μg/mL gentamicin at 37°C in a humidified 5% CO_2_/95% air incubator and passaged them as previously described (Liu et al. 2005).

### DPE preparation

In this study, we used DPE [standard reference material (SRM) 1975], defined by the National Institute of Standards and Technology (NIST; Gaithersburg, MD, USA). SRM 1975 is a dichloromethane extract of the diesel particulate matter SRM 2975, which was generated by a forklift truck using an industrial diesel-powered engine and collected under specifically designed heavy-duty conditions (NIST 2000).

### Exposure to DPE and UVA

Exponentially growing A_L_ cells were trypsinized and replated in 30-mm-diameter petri dishes at 1 × 10^5^ cells/dish for 48 hr, and then treated with either DPE or UVA alone or in combination (DPE + UVA). In the DPE + UVA group, cells were pretreated with DPE in phosphate-buffered saline (PBS) for 30 min and then irradiated with UVA. For UVA radiation, three UV lamps (BLE-IT151, Spectronics Co., Westbury, New York, USA) with an emission wavelength peak at 365 nm were used to irradiate the cells. The culture plates were placed on a table that was 15 cm away from the UV lamps. During UV exposure, the dose rate was simultaneously measured by a radiometer (Photoelectric Instrument Factory of Beijing Normal University, Beijing, China) with a 365-nm detector located the same distance as the culture plates from the UV source.

### Determination of cytotoxicity

After treatment, cultures were washed with PBS, trypsinized, and replated into 100-mm-diameter petri dishes for colony formation. The cultures were incubated for 7 days and then fixed with formaldehyde, stained with Giemsa, and the number of colonies was counted to determine the survival fraction (Xu et al. 2002). We defined the survival fraction as the plating efficiency of treated group divided by the plating efficiency of the control group.

### Quantification of mutations at the CD59 locus and analysis of mutant spectrum

After treatment, we replated cultures in T-75 flasks and cultured them for 5–7 days. This expression period permits surviving cells to recover from the temporary growth lag from DPE and UVA treatment and to multiply such that the progeny of the mutated cells no longer express lethal amounts of the CD59 surface antigen. To determine mutant fractions, 5 × 10^4^ cells were plated into each of six 60-mm dishes in a total of 2 mL of growth medium as previously described (Xu et al. 2002). The cultures were incubated for 2 hr to allow for cell attachment, after which 0.2% CD59 antiserum and 1.5% (vol/vol) freshly thawed complement were added to each dish. The cultures were further incubated for 7–8 days, and then they were fixed, stained, and the number of *CD59*^−^ mutants scored. Controls included identical sets of dishes containing antiserum alone, complement alone, or neither agent. We calculated mutant fractions as the number of surviving colonies divided by the total number of cells plated after correction for any nonspecific killing due to complement alone.

*CD59*^−^ mutants were isolated by cloning and expanded in culture as previously described (Hei et al. 1998). To ensure that all mutants analyzed were independently generated, we isolated only one and, occasionally, no more than two well-separated mutants per dish for analysis. We chose five marker genes located on either the short arm (*WT*, *PTH*, *CAT*, *RAS*) or the long arm (*APO-A1*) of human chromosome 11 for multiplex polymerase chain reaction (PCR) because of their mapping positions relative to the *CD59* gene and the availability of PCR primers for the coding regions of these genes. PCR amplifications were performed for 30 cycles using a DNA thermal cycle model 480 (Perkin-Elmer/Cetus, Waltham, MA, USA) in 20 μL reaction mixture containing 0.2 μg of the EcoRI-digested DNA sample in 1× Stoffel fragment buffer, all four deoxyribo-nucleotide triphosphates (each at 0.2 mM), 3 mM MgCl_2_, 0.2 mM each primer, and 2 U Stoffel fragment enzyme. Each PCR cycle consisted of denaturation at 94°C for 1 min, annealing at 55°C for 1 min, and extension at 72°C for 1 min. After the last cycle, we incubated samples at 72°C for an additional 20 min, electrophoresed them on 3% agarose gels, and stained them with ethidium bromide.

### Apoptosis assay

For detection of apoptosis, we stained cells with Hoechst stain (Kishikawa et al. 2008). After treatment, cells were cultured for 24 hr and then rinsed with PBS twice, and fixed in a 2% paraformaldehyde solution for 15 min at room temperature, and rinsed again three times with PBS. Then the cells were stained with Hoechst 33342 (Sigma, St. Louis, MO, USA) at a final concentration of 5 μg/mL for 20 min at room temperature. We assayed apoptosis under an Olympus 1X71 fluorescence microscope (Olympus, Tokyo, Japan) and considered cells with shrunken, dense morphology and a fragmented nucleus to be apoptotic cells.

### Determination of micronucleus formation

We measured the frequency of micronucleus (MN) formation with the cytokinesis-block technique developed by Fenech and Morley (1986). Briefly, 30 min after treatment, cells were trypsinized and replated into 30-mm petri dishes at a density of 1 × 10^4^ cells. After incubation for 4–6 hr, the growth medium was changed with the medium containing 2.5 μg/mL cytochalasin B (Sigma) and further incubated for 28 hr. Cells were then rinsed with PBS once, and fixed in a 9:1 solution of methanol:acetic acid for 20 min, and rinsed twice with water. The fixed cells were stained with 0.01% (wt/vol) acridine orange for 4 min before observation. MN in the binucleated cells were assayed under an Olympus 1X71 fluorescence microscope and identified morphologically using the criteria of Fenech (1993). At least 1,000 binucleated cells were scored in each experiment for each data point to measure frequency of MN induction.

### Effects of reactive oxygen species quenchers on toxicity of DPE + UVA

We treated exponentially growing A_L_ cells with 20 mM sodium azide (NaN_3_; Sigma), 500 U/mL superoxide dismutase (SOD; Sigma), 20 mM mannitol (Sigma), 500 U/mL catalase (CAT; Sigma), or 1% dimethyl sulfoxide (DMSO; Sigma) with or without concurrent treatment with DPE for 30 min, and then irradiated them with UVA. Then the survival fraction and MN induction were tested as described above. The dose of reactive oxygen species (ROS) quenchers used in the present study was nontoxic and nonmutagenic.

### Electron paramagnetic resonance (EPR) detection of 4-O-TEMPO

To detect 1O_2_, we used the trap probe 2,2,6,6-tetramethyl-4-piperidone hydrochloride (TEMP; purity of 95%). This probe, which has been shown to be specific for 1O_2_ detection (Zang et al. 1995), reacts with 1O_2_ to yield a stable nitroxide radical 4-oxo-2,2,6,6-tetramethyl-piperidine-*N*-oxyl (4-O-TEMPO), having a known three-line EPR spectrum. TEMP (Sigma; 0.05 M) or the stable radical 2,2,6,6-tetramethyl piperidine-*N*oxyl (TEMPO;-10^−6^ M; Sigma) was added to cells 30 min before UVA radiation and the culture medium was collected immediately after radiation. Samples in 25-μL capillaries inserted into 4-mm quartz tubes were used for EPR analysis. EPR spectra were recorded at room temperature on a JEOL JES-FA 200 EPR spectrometer (JEOL, Tokyo, Japan). The measurements were repeated at least three times for each sample. We set the microwave source of the EPR at 9.0 GHz and the power at 3.0 mW. Modulation frequency and modulation amplitude were 100 kHz and 0.1 mT, respectively. The time constant was 0.3 sec, and scan time was 120 sec. The relative signal intensity of 4-O-TEMPO is represented by dividing the ratio of the 4-O-TEMPO signal intensity of the treated group by that of the control group.

### Data analysis

All values were expressed as means ± SD. We tested significant differences at the *p* < 0.01 level using analysis of variance followed by Dunnett *t*-tests or two-tailed Student *t*-tests.

## Results

### Lethality of DPE and UVA in A_L_ cells

**Figure 1:**
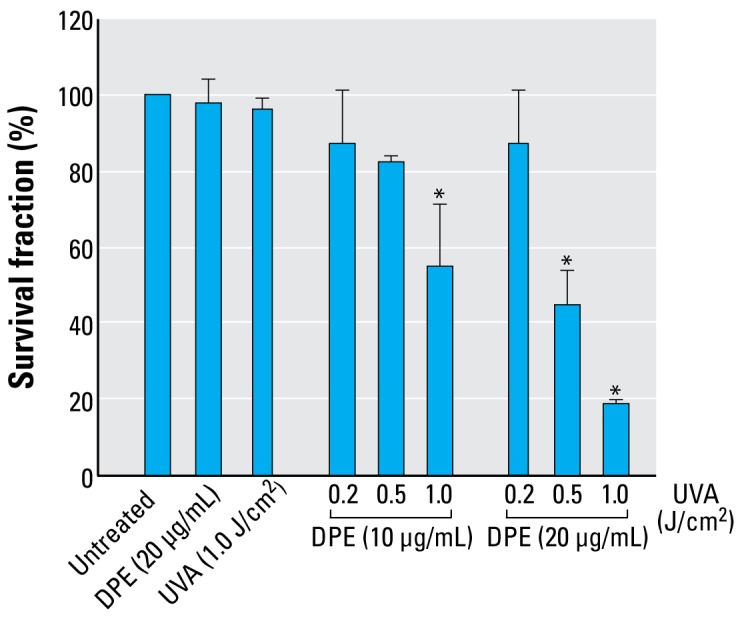
Effects of DPE, UVA, or DPE + UVA on surviving fraction in A_L_ cells (mean + SD): pooled data from three independent experiments. *p < 0.01 significant between combined treated group and singly treated group.

The survival fractions of A_L_ cells treated with DPE (10–20 μg/mL) and/or UVA (0.2–1.0 J/cm^2^) were determined by colony formation assay. The normal plating efficiency of A_L_ cells used in the present study was about 80%. As shown in Figure 1, single treatment with DPE (20 μg/mL) or UVA (1.0 J/cm^2^) slightly changed the survival fractions of A_L_ cells. However, with the cotreatment of DPE and UVA, the survival fractions of A_L_ cells showed a dose-dependent decrease. For example, at doses of 10 μg/mL DPE + 1.0 J/cm^2^ UVA, 20 μg/mL DPE + 0.5 J/cm^2^ UVA, and 20 μg/mL DPE + 1.0 J/cm^2^ UVA, the survival fractions were significantly decreased to 54.87 ± 16.6%, 44.6 ± 8.97%, and 18.43 ± 1.56%, respectively, compared with the untreated group (*p* < 0.01).

### Mutation frequencies at CD59 gene and mutant spectra

**Figure 2:**
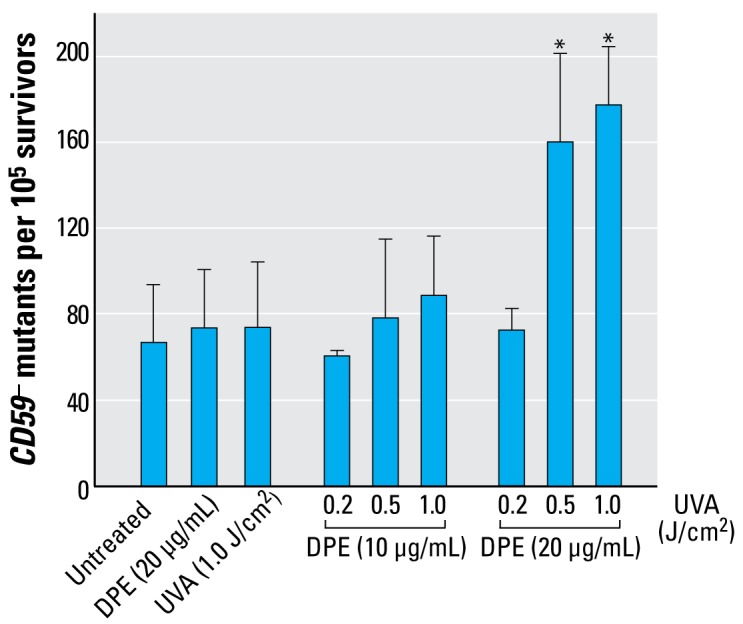
Induction of *CD59*^−^ mutant fractions per 10^5^ survivors in A_L_ cells (mean + SD): pooled data from three independent experiments. *p < 0.01.

The average background mutant fraction of A_L_ cells was about 67 ± 27 mutants per 10^5^ survivors. As shown in Figure 2, the mutation fractions induced by DPE (20 μg/mL) or UVA (0.5 J/cm^2^) alone were 73 ± 27 and 73 ± 20 mutants per 10^5^ survivors, respectively. However, with the cotreatment of DPE and UVA, the mutation fractions of A_L_ cells showed a dose-dependent increase. For example, at doses of 20 μg/mL DPE + 0.5 J/cm^2^ UVA and 20 μg/mL DPE + 1.0 J/cm^2^ UVA, the mutation yield at the *CD59* locus was dramatically increased to 161 ± 41% and 177 ± 27% (*p* < 0.01).

**Figure 3:**
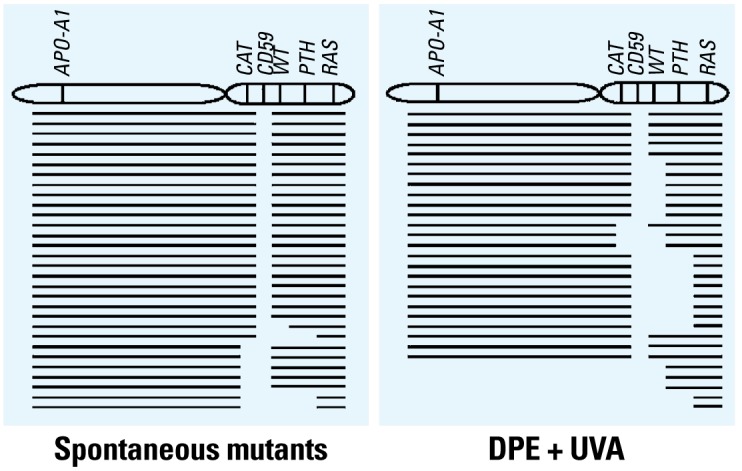
Mutational spectra of *CD59* mutants either of spontaneous origin or from cells exposed to DPE (20 μg/mL) + UVA (0.5 J/cm^2^), determined by multiplex PCR. Each line represents the spectrum for a single, independent mutant. Blank spaces indicate missing markers.

To compare the type and size of mutations either of spontaneous origin or induced by DPE + UVA treatment, multiplex PCR and primer sequences for five marker genes (*WT*, *PTH*, *CAT*, *RAS*, and *APO-A1*) were used as previously described (Hei et al. 1998). As shown in Table 1 and Figure 3, most spontaneous *CD59*^−^ mutants (21 of 30, 70.0%) showed no detectable changes in any of the marker genes examined. In contrast, only 4 of 30 (13.3%) of mutants derived from cells exposed to DPE (20 μg/mL) + UVA (0.5 J/ cm^2^) retained all the marker genes examined, whereas 26 of 30 (86.7%) of the mutants had lost at least one additional marker, which included 8 of 30 (26.7%) that lost the proximal *APO-A1* located on the long arm of the chromosome. These results indicated that DPE + UVA easily induced multilocus deletions.

### Induction of apoptosis in A_L_ cells

**Figure 4:**
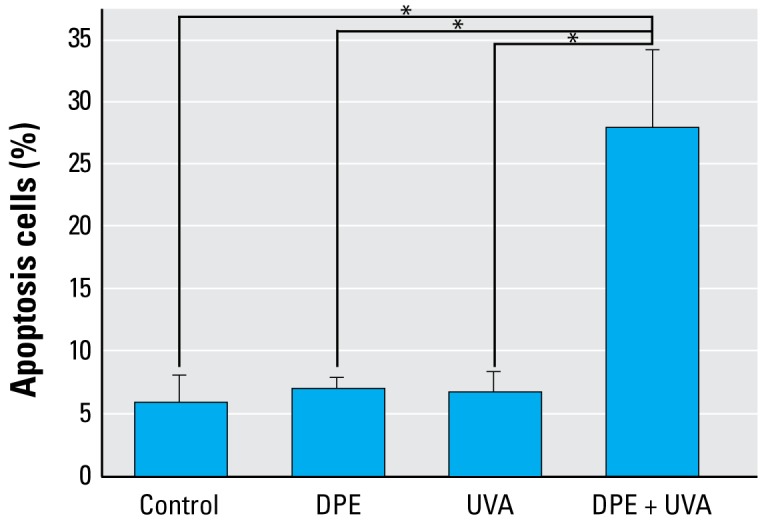
Apoptotic A_L_ cells induced by DPE, UVA, or DPE + UVA (mean + SD): pooled data from three independent experiments. *p < 0.01.

Figure 4 shows the percentage of cells with apoptotic morphology after treatments. In control A_L_ cells, 5.9 ± 2.3% were apoptotic. The apoptosis fractions induced by treatment with either DPE (20 μg/mL) or UVA (0.5 J/cm^2^) alone were 7.0 ± 1.0% and 6.9 ± 1.6%, respectively. However, when the cells were treated with DPE (20 μg/mL) + UVA (0.5 J/cm^2^), the fraction of apoptotic cells was remarkably increased, from 5.9 ± 2.3% to 28.0 ± 6.1% (*p*< 0.01).

### Induction of MN in A_L_ cells

**Figure 5:**
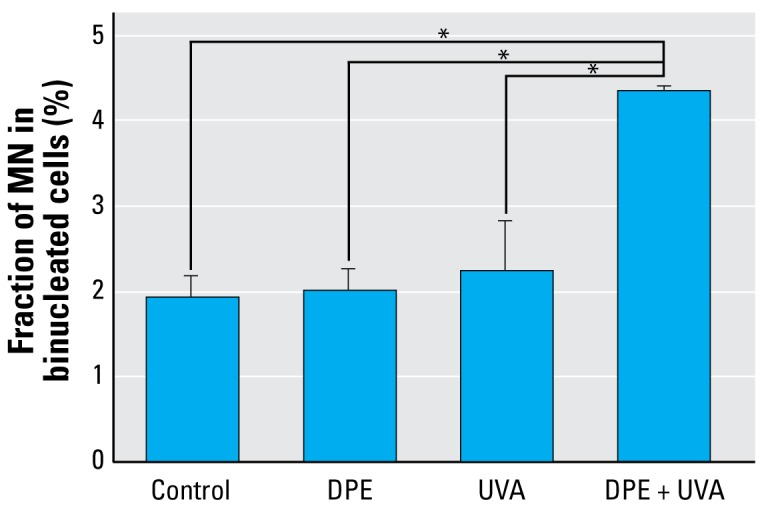
Effects of DPE, UVA, or DPE + UVA on MN formation in A_L_ cells (mean + SD): pooled data from three independent experiments. *p < 0.01.

Figure 5 shows the fractions of binucleated cells with MN. The average fraction of cells with MN in the control group was 1.92 ± 0.25%. The MN fractions induced by DPE (20 μg/mL) or UVA (0.5 J/cm^2^) alone were 2.01 ± 0.25% and 2.24 ± 0.58%, respectively. A significant increase of MN induction was observed in the DPE (20 μg/mL) + UVA (0.5 J/cm^2^) treatment group that was more than twice as high as in the controls (*p* < 0.01).

### Effects of ROS quenchers on survival fraction and MN induction

**Figure 6:**
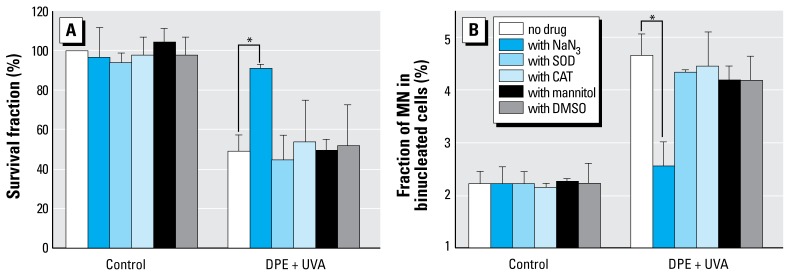
Effects of ROS quenchers on DPE + UV induced survival fraction (*A*) and MN formation (*B*) in A_L_ cells (mean + SD). Data were pooled from three independent experiments. *p < 0.01.

We used NaN_3_, CAT, SOD, mannitol, and DMSO to determine the role of ROS in the DPE + UVA–induced cytotoxicity and genotoxicity. As shown in Figure 6, CAT, SOD, mannitol, or DMSO had little protective effect on DPE (20 μg/mL) + UVA (0.5 J/cm^2^) caused cytotoxicity and genotoxicity. NaN_3_, a specific 1O_2_ scavenger, effectively protected cells from DPE + UVA–induced cell damage and DNA damage. In the presence of NaN_3_, the survival fraction of A_L_ cells treated with DPE + UVA increased from 49.13 ± 8.00% to 91.37 ± 1.81% (*p* < 0.01; Figure 6A), and MN induction in A_L_ cells significantly decreased from 4.7 ± 0.4% to 2.5 ± 0.4% (*p* < 0.01; Figure 6B).

### Determination of 1O_2_ in the medium of cells treated with DPE + UVA

**Figure 7:**
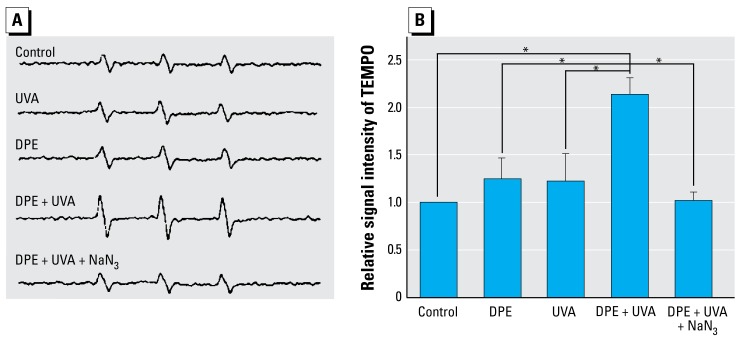
Detection of the increase in the 4-O-TEMPO signal in A_L_ cells. Data were pooled from three independent experiments. (*A*) Three-line EPR spectra of 4-O-TEMPO signal. (*B*) Relative signal intensity of 4-O-TEMPO (mean + SD). *p < 0.01.

As shown in Figure 7A, 4-O-TEMPO triplet spectra increased in cells treated with DPE (20 μg/ mL) + UVA (0.5 J/cm^2^), and NaN_3_ (20 mM) significantly reduced this signal. DPE alone and UVA radiation alone did not notably change the signal of 4-O-TEMPO. As shown in Figure 7B, relative signal intensity of 4-O-TEMPO was significantly increased in cells treated with DPE + UVA (*p* < 0.01), by about 1.1-fold higher than the untreated group. In addition, NaN_3_ efficiently decreased the relative signal intensity of 4-O-TEMPO induced by DPE and UVA to nearly the same as the control group.

## Discussion

Both diesel exhaust and UV radiation are ubiquitous in the environment. Most Ames tests have demonstrated that DEPs and DPEs are mutagenic and are closely related to the generation of ROS (DeMarini et al. 2004; Pohjola et al. 2003; Tsurudome et al. 1999). At doses greater than 100 μg/mL, Li and colleagues found that the organic extracts of DEPs were able to generate ROS and induce cell death and apoptosis in macrophages (Hiura et al. 1999; Li et al. 2002). Our previous work also demonstrated the mutagenicity of DEPs in mammalian cells (Bao et al. 2007). However, there is limited evidence on the effects of UVA on the genotoxicity of DPE, especially at low-dose exposures. In the present study in human–hamster hybrid (A_L_) cells, we found that coexposure of DPE and UVA radiation severely decreased survival fractions and greatly enhanced cellular apoptosis, mutation fractions, and MN induction even though the treatments of cells with either UVA or DPE alone induced minimal cytotoxicity and genotoxicity. UVA or diesel exhaust exposure alone is most likely to cause single-base substitutions or less frequent insertions, deletions, and multiple-base substitutions/deletions, respectively (Besaratinia et al. 2004; Sato et al. 2000). By analyzing the mutation spectrum on chromosome 11 of A_L_ cells, we found that treatment with DPE + UVA tended to cause multilocus deletions, which is similar to the DNA damage produced by ionizing radiation, suggesting that exposure to DPE + UVA has a risk of cancer similar to that of ionizing radiation (Hei et al. 1997). This result might indicate the different mutagenic mechanisms between single treatment by DPE/UVA and their coexposure.

DEPs are heterogeneous, consisting of more than 450 different organic compounds, including xenobiotics such as PAHs, halogenated aromatic hydrocarbons, and redox-active quinones (Athanasiou et al. 1987; Li et al. 2002; Whong et al. 1981). Some of the individual components are cytotoxic and mutagenic in mammalian cells, but most are promutagens that require activation to electrophilic metabolites to exert their mutagenic or carcinogenic effects (Xue and Warshawsky 2005). Some studies have demonstrated an increase in direct-acting mutagens in air samples collected in the summer months, which suggests that photochemical reactions might activate air samples (e.g., DPE) to be more mutagenic (Casellas et al. 1995; Clunies-Ross et al. 1996). Photoirradiation has been found to enhance both mutagenicity and cytotoxicity of chemicals, such as azido analogues of amsacrine and other 9-anilinoacridines (Iwamoto et al. 1992), whereas other studies have shown that an exposure to near-UV light converted the promutagens, including PAHs, into direct-acting mutagens (Barnhart and Cox 1980; de Wiest et al. 1982). These findings suggest that sunlight, especially UV, as a major modifying factor played an essential role in the adverse health effects induced by chemical pollutions, such as PAH, DPE, and organic extracts of urban particulates. In the present study, treatment with UVA and DPE alone or UVA radiation followed by DPE exposure (data not shown) had slight toxic effects on A_L_ cells, which indicates that DPE might be photosensitized by UVA, leading to an enhancement of cellular and genomic damage in mammalian cells.

There are two possible ways to activate the organic compounds of DPE, such as PAHs, to become toxic and carcinogenic. One way is metabolic activation. Metabolic products, such as diol epoxides and diones, are highly carcinogenic and induce covalent DNA adducts and oxidative DNA lesions (Ohnishi and Kawanishi 2002; Sims et al. 1974). Metabolic activated xenobiotic in DPE can also exert stimulatory or toxic effects via the generation of ROS (Ichinose et al. 1997; Kumagai et al. 1997; Park et al. 1996; Pinkus et al. 1996). Another way is photoactivation. It is possible that after absorbance of UVA energy, xenobiotic molecules in DPE, especially PAHs, are elevated from the ground state to an excited state. The excited PAHs can react directly with biological molecules (type I) or can react with triplet-state oxygen to form excited singlet-state oxygen (major) or other ROS (minor; type II) (Toyooka and Ibuki 2006). 1O_2_ has been shown to play an important role in cellular and DNA damage induced by coexposure to PAHs and UVA (Ibuki et al. 2002; Liu et al. 1999; Toyooka et al. 2006). 1O_2_ can produce single-strand breaks in cell-free DNA and oxidative DNA base modifications (Schulz et al. 2000; Yang et al. 1999). These DNA lesions may inevitably contribute to the mutation induction and MN formation. Using NaN_3_, which is widely used as an efficient 1O_2_ quencher (Li et al. 2001; Sparrow et al. 2002, 2003), we found that the decrease in survival fraction and MN generation by DPE + UVA exposure were effectively inhibited, whereas the ROS quenchers CAT, SOD, mannitol, and DMSO had little effect. Furthermore, using a 1O_2_ trapping probe, TEMP, coupled with EPR spectroscopy, we found enhanced production of 1O_2_ in the DPE + UVA exposure group, but not in the groups treated with DPE or UVA alone. These results indicate that photoactive production of 1O_2_ is mainly involved in the process of UVA activated toxicity of DPE in mammalian cells.

In summary, our study provides direct evidence of the augmented cytotoxicity and genotoxicity of DPE activated by UVA through the photoactive production of 1O_2_. Because increasing amounts of diesel exhaust have been released into the environment and the depletion of ozone layer has led to the more UV exposure for humans, it is important to determine whether diesel exhaust may synergize with UVA radiation to amplify genetic damage. The underlying mechanisms of these synergistic effects need to be further elucidated both *in vitro* and *in vivo*.

## Figures and Tables

**Table 1:** Frequencies of mutations of spontaneous origin or induced by DPE + UVA, as determined by multiplex PCR analyses: *CD59*^−^ with or without at least one additional marker.

		*CD59*^−^ only		*CD59*^−^ plus at least one other marker	
Group	Total mutants	No.	%	No.	%
Spontaneous	30	21	70	9	30
DPE (20 μg/mL) + UVA (0.5 J/cm^−^ )	30	4	13.3	26	86.7
